# Spontaneous Subclavian Artery Dissection: A Case Report and Narrative Review

**DOI:** 10.7759/cureus.97110

**Published:** 2025-11-17

**Authors:** Katerina Sidiropoulou, Panagiotis M Chatzipanagiotis, Dimitrios Karamanos, Konstantinos Tigkiropoulos

**Affiliations:** 1 First Surgical Department, Papageorgiou General Hospital, Aristotle University, Thessaloniki, GRC; 2 Radiology Department, Papageorgiou General Hospital, Aristotle University, Thessaloniki, GRC

**Keywords:** isolated subclavian artery dissection, narrative review, spontaneous dissection, subclavian artery, upper arm ischemia

## Abstract

Spontaneous subclavian artery dissection (SSAD) is a rare vascular entity. Its clinical presentation varies significantly, ranging from asymptomatic cases to major neurological events, such as stroke or upper limb ischemia. We present a case of SSAD managed conservatively in a patient who presented with acute upper limb ischemia. A 59-year-old male presented to the emergency department with a 2-hour history of left upper limb pain, with minor motor and sensory deficit, and absence of palpable pulses. Duplex ultrasound revealed an intimal flap in the subclavian artery, while computed tomography angiography (CTA) demonstrated a dissection at the origin of the left subclavian artery that extended to the axillary artery, with distal perfusion impairment. Initial intravenous administration of unfractionated heparin, followed by therapeutic low-molecular-weight heparin during hospitalization, led to improvement in limb perfusion and restoration of palpable pulses. In addition, we provide a narrative review of the literature on SSAD. A search of PubMed, Scopus, Embase, and Google Scholar up to July 2025 identified 18 published case reports describing clinical presentation and treatment strategies. Spontaneous subclavian artery dissection is a rare vascular condition with diverse clinical manifestations and therapeutic approaches. Computed tomography angiography remains the primary diagnostic imaging modality in conjunction with clinical evaluation. Given the limited number of reported cases, there is currently no consensus on optimal management, and treatment should be individualized based on the patient’s clinical presentation.

## Introduction

Isolated subclavian artery dissection is a rare vascular condition and is usually related to blunt trauma, arterial catheterization, or connective tissue disorders. Spontaneous isolated subclavian artery dissections (SSADs) are an even rarer entity, with only a few cases reported in the literature. As the subclavian artery supplies the upper limb and part of the cerebral circulation through the vertebral artery, its dissection may cause a wide range of symptoms. Clinical presentation usually includes sudden onset of back and chest pain, upper limb ischemia, and stroke [1-4]. We report the conservative management of a patient with upper limb ischemia caused by SSAD. Additionally, due to the rarity of this vascular entity, a narrative review of the literature was also performed.

## Case presentation

**Figure 1:**
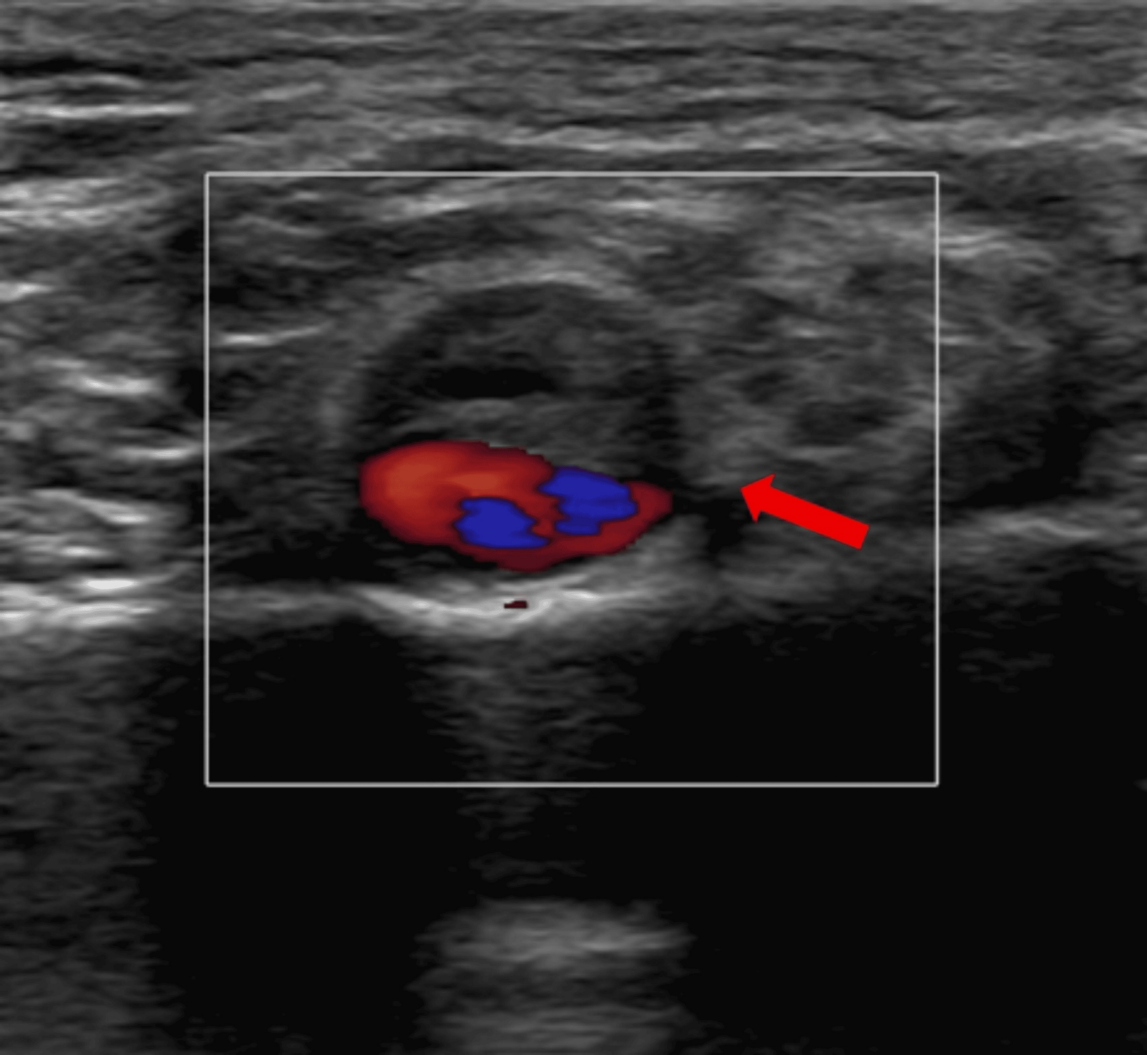
Axillary artery dissection with intimal flap (arrow) and thrombosis of the false lumen on duplex ultrasound.

A 59-year-old Caucasian male presented to our emergency department with a 2-hour history of sudden left upper limb pain, pallor, and motor and sensory deficits. The patient had no significant past medical history, and no prior exercise or trauma was mentioned. Shortly after the event, he self-administered 100 mg of aspirin at home with subsequent improvement of symptoms. He denied chest pain before or after the onset of symptoms. On clinical examination, the patient was hemodynamically stable; arterial blood pressure was 135/89 mmHg, and heart rate was 83 beats per minute. He had mild motor and sensory deficits. His fingers were pale, and no radial or brachial pulse was palpable. The limb was classified as Rutherford grade IIa. Colored duplex ultrasound of the left upper arm showed the presence of an intimal flap in the subclavian, axillary, and brachial artery, with significant stenosis due to thrombosis of the false lumen (Figure 1).

**Figure 2:**
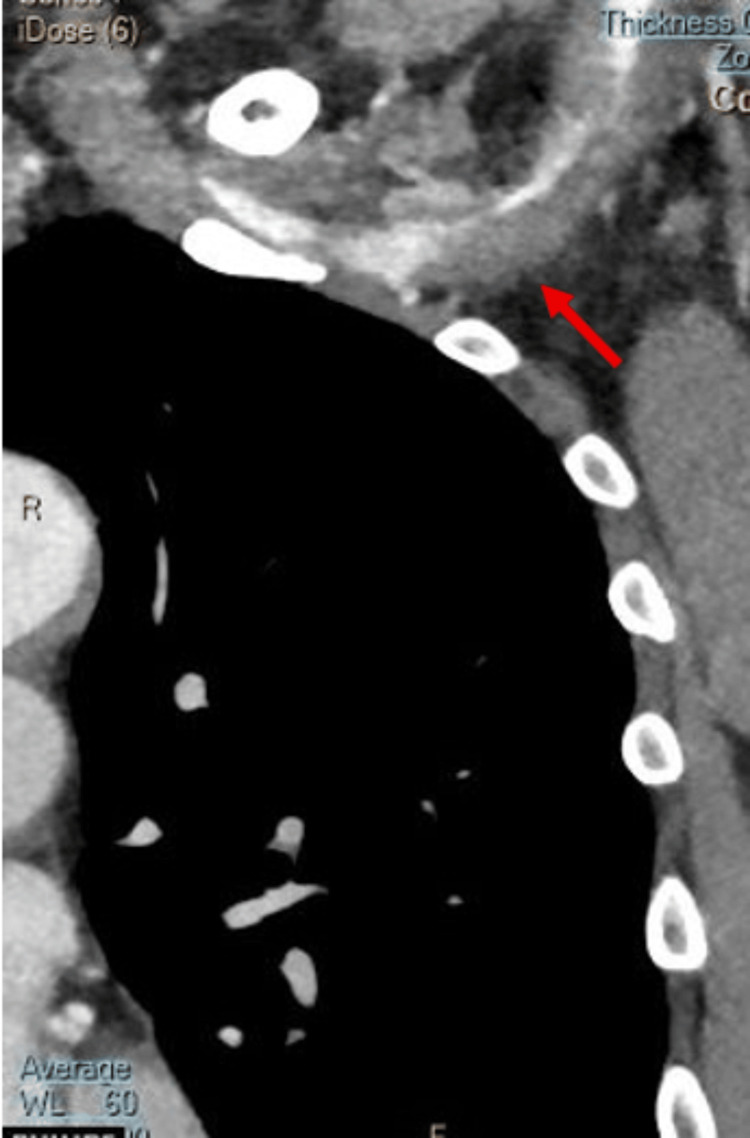
Subclavian artery dissection with thrombosis of the false lumen in the sagittal plane (arrow).

**Figure 3:**
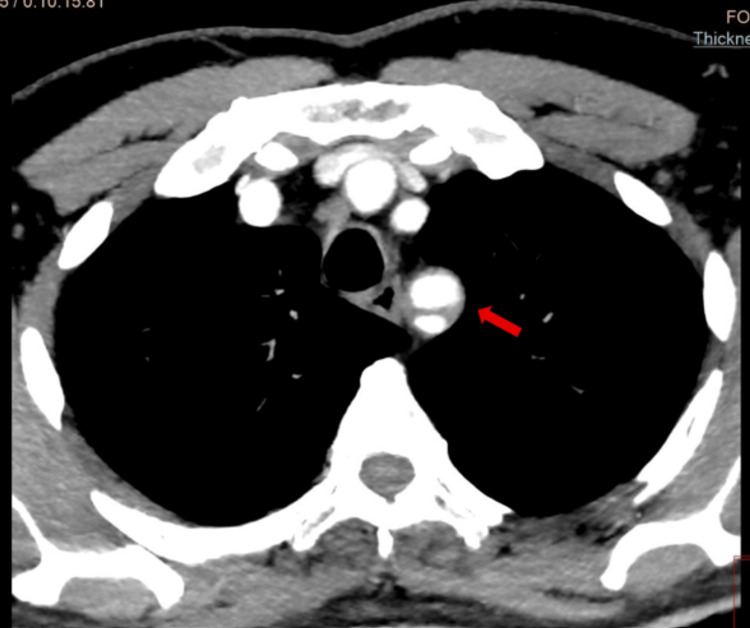
Dissection of the left subclavian artery (arrow) on CT angiography.

A computed tomography angiography (CTA) of the aorta and upper limb was subsequently performed to exclude an aortic dissection. It demonstrated a dissection at the origin of the left subclavian artery extending to the brachial artery, with distal perfusion impairment (Figures 2, 3). No pathological findings were observed in the aorta or the left common carotid artery.

The patient was treated initially with intravenous administration of unfractionated heparin (75 IU/kg), followed by low molecular weight heparin (LMWH) in a therapeutic dose. Due to improved perfusion of the left upper limb, conservative management was chosen. The patient had palpable pulses in the left brachial and radial artery 18 hours after the administration of heparin, and no motor or sensory deficits were present. He remained hemodynamically stable. He was discharged after three days of hospitalization with lifelong intake of aspirin 100 mg daily and LMWH in prophylactic dose for one month.

**Figure 4:**
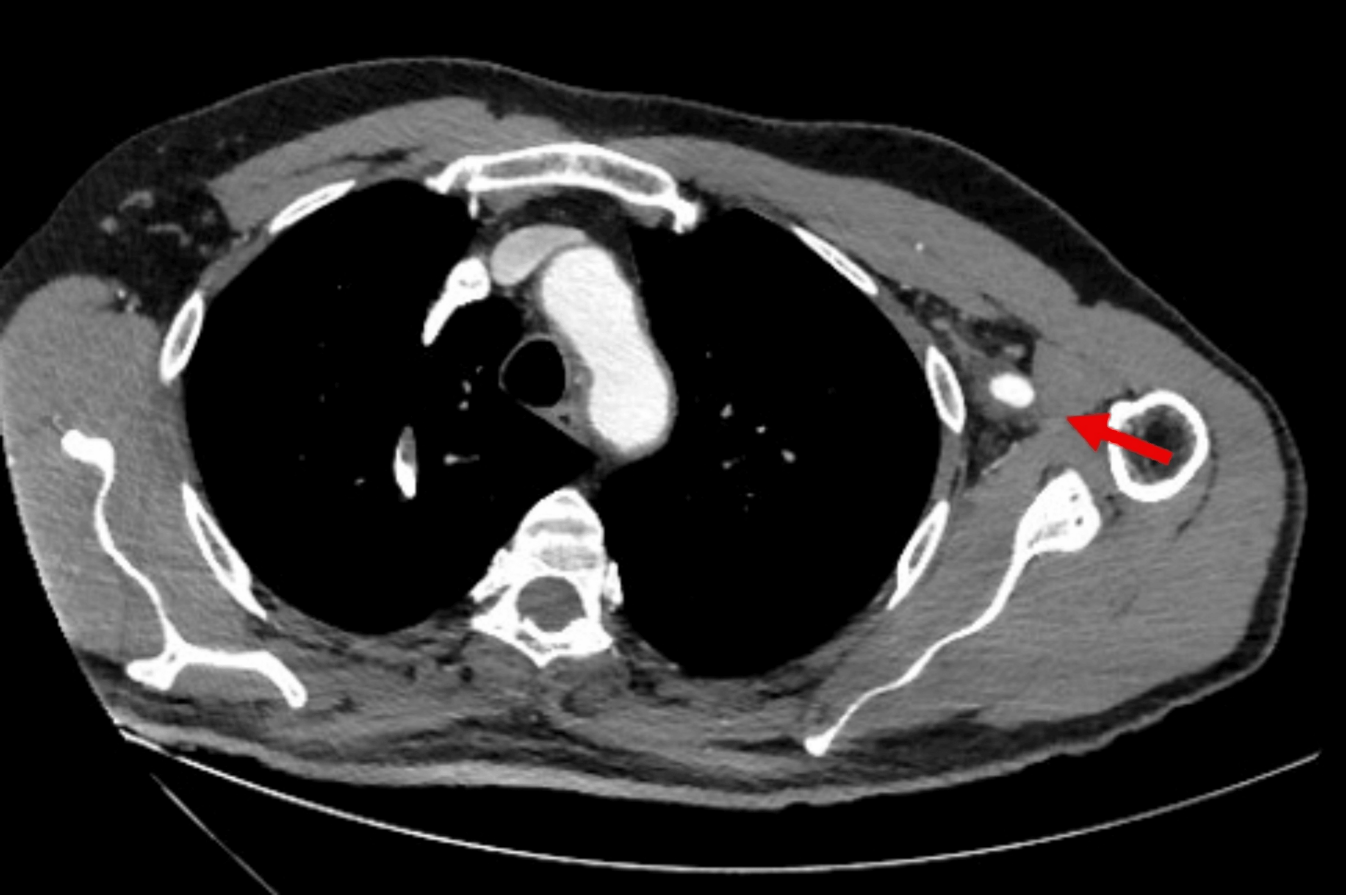
Restoration of lumen patency of the left axillary artery (arrow) on CTA at one month. CTA: computed tomography angiography

**Figure 5:**
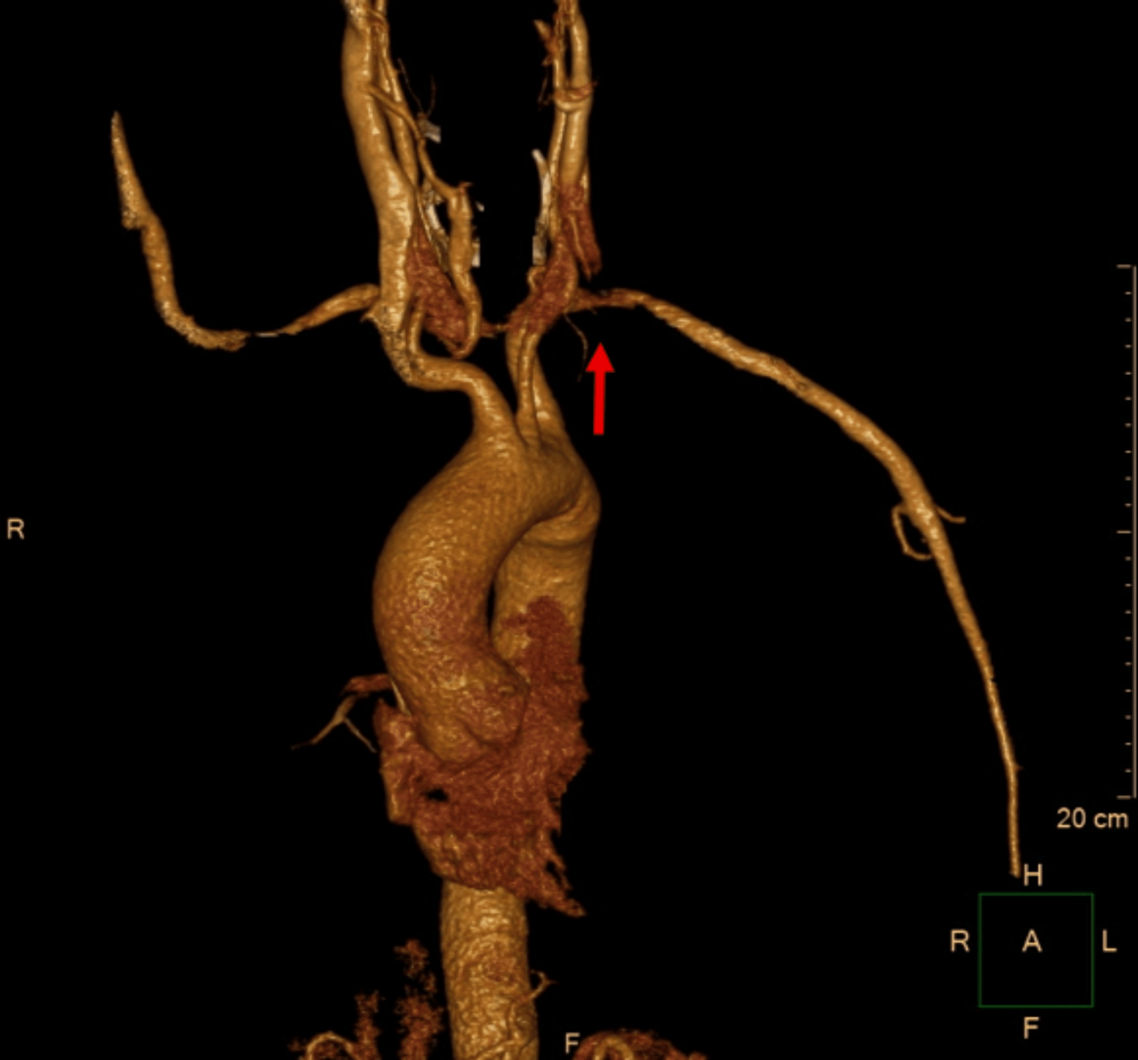
Stenosis of left subclavian artery (arrow) in CT angiography 30 days after the event.

A CT angiography of the aorta and left upper limb was performed 30 days after discharge. A moderate stenosis at the origin of the left subclavian artery was found, attributed to thrombosis of the false lumen, while in the axillary and brachial artery, a significant retraction of the thrombosed false lumen was shown (Figures 4, 5). On clinical examination, the patient had palpable pulses in the radial artery and experienced no symptoms during the follow-up period.

## Discussion

We conducted a narrative review due to the limited number of case reports and the scarcity of available data. The search was conducted by two authors independently for records published up until July 2025 in PubMed, MEDLINE, Embase, Scopus, and Google Scholar.

This review included full-text articles in the English language. Eligibility criteria were case reports or case series of spontaneous, isolated subclavian artery dissections. Exclusion criteria were subclavian artery aneurysm dissection, aortic dissection, cadaveric reports, and subclavian artery dissection due to blunt trauma or medical procedures. Duplicate records, animal studies, articles reporting spontaneous dissection of arteries other than the subclavian artery, and studies deemed irrelevant were also excluded by automation tools and researchers. After initial selection, full-text articles were further evaluated for eligibility by the researchers.

**Figure 6:**
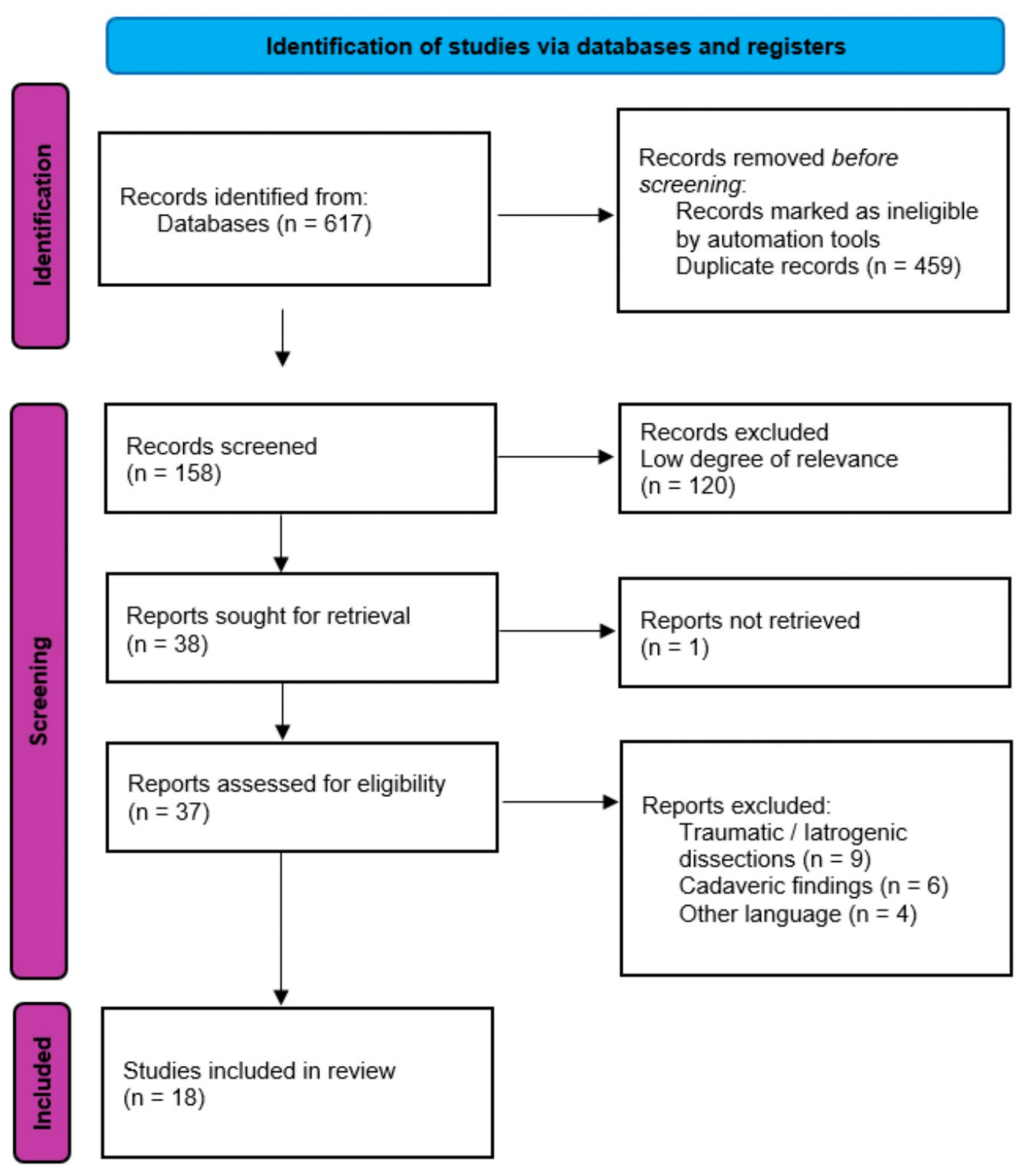
PRISMA 2020 flow diagram. PRISMA: Preferred Reporting Items for Systematic Reviews and Meta-Analyses

Initially, 617 records were identified from the databases. The Preferred Reporting Items for Systematic Reviews and Meta-Analyses (PRISMA) 2020 flow diagram describes the exclusion and selection process of the articles. A total of 18 articles met the eligibility criteria (Figure 6). Table 1 summarizes all the cases, demographics, clinical presentation, treatment, and outcome.

**Table 1:** List of published articles regarding spontaneous subclavian artery dissection. NSTEMI: non-ST-segment elevation myocardial infarction; BE: balloon-expandable; SE: self-expanding; UL: upper limb; N/R: not reported; LL: lower limb

Studies (year)	Gender	Age (years)	Side	Clinical presentation	Treatment/antithrombotic treatment	Follow-up
Garewal and Selhorst (2005) [4]	M	54	Left	Stroke (left hemispheric infarct, right cerebellar infarct, C4-C5 spinal cord ischemia), mild UL ischemia	Conservative/initially anticoagulation and then switched to aspirin	Six months; complete remission of symptoms
Nagino et al. (2017) [5]	M	58	Left	Left arm paresthesia, chest and neck pain, stroke (cerebellum and medulla infarction)	Conservative blood pressure control/no anticoagulation	One month; full remission of symptoms
Ananthakrishnan et al. (2009) [6]	F	62	Left	UL ischemia	Endovascular therapy with BE non-covered stent/double antiplatelet therapy	Two months; no complications
Funada et al. (2010) [7]	F	58	Left	Mild UL ischemia	Conservative/antiplatelet therapy	Two weeks; no complications
Fernandes et al (2010) [8]	F	31	Left	Stroke (bilateral infarction of cerebellum-occipital lobes)	N/R	N/R
Nakamura et al. (2010) [9]	F	41	Left	Mild UL ischemia, bilateral lower limb ischemia	Bilateral thrombectomy of LL, Anticoagulation+antiplatelet therapy	Three months; resolution of thrombus, Two years; no more embolic events
Winblad et al. (2012) [10]	M	54	Left	Stroke (left cerebellar, right occipital)	Conservative/anticoagulation with Coumadin	Three months; asymptomatic
Marik and Mclaughlin (2013) [1]	F	36	Left	Neck pain, left shoulder pain, hypertensive urgency	Conservative, blood pressure management/anticoagulation N/R	N/R
Touchan et al. (2014) [11]	F	55	Left	Symptoms mimicking NSTEMI, chest pain, nausea, hypertensive urgency	Endovascular therapy with non-covered BE stent/anticoagulation N/R	N/R
De Bruyne et al. (2015) [12]	M	73	Right	UL ischemia	Conservative/anticoagulation with warfarin	29 months, no complications
Jeon and Cho (2017) [13]	M	71	Left	Stroke (medulla-cerebellar infarction)	Conservative/initially single antiplatelet therapy, after worsening of symptoms, double antiplatelet therapy	Six months, remaining left-sided hypesthesia
Atere et al. (2020) [14]	F	61	Left	Chest pain radiating to neck and left arm	Conservative, blood pressure management/single antiplatelet therapy	N/R
Williams et al. (2020) [15]	F	65	Left	Asymptomatic, incidental finding	Conservative/anticoagulation N/R	N/R
Kim (2021) [16]	F	72	Left	Chest pain, stroke (right hemiparesis, dysarthria)	IV thrombolysis and then double antiplatelet treatment	N/R
Alloush et al. (2022) [17]	F	61	Left	UL ischemia, stroke (cerebellar infarction)	Thrombectomy of upper arm, nerve block, and chemical sympathectomy/N/R	N/R
Yanamadala and Salman (2022) [18]	F	78	Left	Asymptomatic, incidental finding	Conservative/anticoagulation N/R	One month, asymptomatic
Dalai et al. (2023) [19]	F	50	Right	UL ischemia	Endovascular thromboaspiration, catheter-directed thrombolysis, non-covered SE stent/double antiplatelet therapy	Six months, no complications
Weidmayer and Liao (2024) [20]	M	71	Right	Horner syndrome	Conservative/single antiplatelet therapy	N/R

Female patients were 66.6% (n=12), and the mean age was 58.4 years. The left subclavian artery was mostly affected, 83.3% (n=15). Eight patients (44.4%) presented with upper limb ischemia, while six patients had stroke symptoms due to thrombosis of the vertebral artery. Conservative management with antiplatelet or anticoagulant therapy was preferred in 72.2% (n=13) of the patients. Endovascular stent placement was performed in three patients, mechanical thrombectomy in one patient, while in one case, management is not reported. In 50% of the cases, no complications were reported in follow-up. One patient had residual left-sided hypesthesia at six months, and in eight cases, follow-up information is not reported.

Isolated subclavian artery dissections are rare and most commonly result from trauma [21], iatrogenic injury after arterial catheterization [22], anomalies in the aortic arch [23], or other uncommon conditions such as arteritis, fibromuscular dysplasia, and neurofibromatosis [19]. The exact pathogenesis of spontaneous subclavian artery dissection remains unclear; however, atherosclerosis in conjunction with arterial hypertension appears to play an important role [3,9]. In our study, 55% of patients had a history of arterial hypertension. Moreover, there appears to be a possible female sex prevalence, as suggested by our review and by forensic reports of SSAD [24,25].

Clinical symptoms may include upper limb ischemia, severe pain, pulselessness, paresthesia, and pallor [5-7,12,17,19]. Patients may also present with chest, back, or neck pain mimicking myocardial infarction, or the condition may be entirely asymptomatic [1,5,11,14,15,18]. Involvement or thrombosis of the left vertebral artery may lead to ischemic stroke manifestations, such as hemiparesis, nausea, vomiting, dysarthria, and visual disturbances [4,5,8,10,13,16,17]. Initial diagnosis can be suggested by duplex ultrasound; however, CTA should be performed to confirm the diagnosis, delineate the full extent of the dissection, and assess suitability for potential endovascular treatment [1,10,13].

Conservative management with antiplatelet or anticoagulant therapy, particularly in cases with thrombosis of the false lumen, has been associated with good outcomes and complete remission of symptoms [1,4,5,7,10,12-16,18,20]. In our case, anticoagulation with low-molecular-weight heparin (LMWH) and lifelong intake of aspirin were selected to prevent thrombus propagation and maintain distal perfusion.

Endovascular treatment with non-covered balloon-expandable or self-expanding stents has been reported in three cases with no postoperative complications during follow-up [6,11,19]. In one case, Alloush et al. reported successful management with thrombectomy of the upper arm combined with nerve block and chemical sympathectomy [17].

Although the number of published cases remains limited, some common features can be found. Most patients were middle-aged and had a medical history of arterial hypertension, which may be a predisposing factor, while the left subclavian artery appears to be more frequently affected. Both conservative and endovascular management have resulted in favorable outcomes, highlighting that timely diagnosis and individualized treatment likely play a greater role in recovery than a specific therapeutic method. Additional case reports will be essential to better understand this rare vascular disease.

## Conclusions

Although spontaneous subclavian artery dissection is an uncommon pathology, increased awareness and early recognition are essential to ensure timely management and minimize complications. CTA remains the imaging modality of choice for diagnosis, and while there is no clear consensus on the optimal treatment, both endovascular treatment and conservative management have shown similarly favorable outcomes when individualized according to clinical presentation. Our case further supports that conservative management can be a safe and effective option even in patients presenting with acute upper limb ischemia, provided that distal perfusion is maintained and the patient is closely monitored. But a longer follow-up period would also be valuable to confirm long-term vessel patency.
